# Mediating role of self-efficacy in the relationship between social integration and self-disgust among patients with stroke

**DOI:** 10.3389/fneur.2026.1868193

**Published:** 2026-09-11

**Authors:** Cai-Dan Liu, Huan-Wen Luo, Yan-Lan Yu, Yun-Bo Zhou, Jia-Yi Zeng

**Affiliations:** 1 Department of Neurology, The First Affiliated Hospital of Hunan University of Chinese Medicine, Changsha, Hunan Province, China; 2 Nursing department, The First Affiliated Hospital of Hunan University of Chinese Medicine, Changsha, Hunan Province, China; 3 Hunan University of Chinese Medicine, Changsha, Hunan Province, China

**Keywords:** mediating effect, mental health, self-disgust, self-efficacy, social integration, stroke

## Abstract

**Objective:**

This study examined the mediating role of self-efficacy in the relationship between social integration and self-disgust among patients with stroke, with the aim of informing the development of targeted interventions.

**Methods:**

In this cross-sectional study, a total of 207 patients with stroke completed a general demographic questionnaire, the Stroke Rehabilitation Self-Efficacy Assessment Scale, the Social Network Scale, and a self-disgust assessment tool. Pearson's correlation analyses were performed to examine the relationships among self-efficacy, social integration, and self-disgust. Mediation analysis was then conducted, with the significance of indirect effects evaluated using the bootstrap method.

**Results:**

Median (interquartile range) scores for self-disgust, social integration, and self-efficacy were 40 (36, 45), 3 (2, 5), and 70 (67, 77) respectively. Correlation analysis indicated that self-disgust was significantly negatively correlated with social integration (*p* < 0.01), and with self-efficacy (*p* < 0.01). Additionally, social integration was significantly positively correlated with self-efficacy (*p* < 0.01). Mediation analysis demonstrated that self-efficacy exerted a significant indirect effect on the relationship between social integration and self-disgust (effect = −0.475), with the indirect pathway through self-efficacy was associated with 43.42% of the total observed effect.

**Conclusion:**

Our results suggest that self-efficacy may partially mediate the relationship between social integration and self-disgust in patients with stroke. Interventions that enhance self-efficacy and promote social integration may reduce self-disgust and support rehabilitation outcomes.

## 1 Introduction

Stroke is an acute cerebrovascular event characterized by the sudden occlusion or rupture of cerebral vessels, resulting in ischemia and hypoxia of localized brain tissue and subsequent neurological deficits (1). Epidemiological studies have confirmed that stroke is a leading cause of disability and mortality worldwide (2). Following stroke, many patients experience permanent functional impairments, including limb hemiplegia, language impairment, and abnormal facial expressions. These deficits often leave patients dependent on others for daily care and may result in the sudden loss of previous family and social roles. Combined with changes in body image and limitations in mobility and communication, such challenges can easily lead to feelings of stigma, worthlessness, and social withdrawal (3). As a result, patients may develop negative perceptions and rejection toward their impaired bodies, disabled behaviors, and declining social functioning, thereby contributing to the development of self-disgust as a maladaptive cognitive and emotional response.

Self-disgust is a persistent negative self-perception encompassing aversion to one's own appearance (e.g., perceiving oneself as repulsive) and rejection of one's behavioral patterns (e.g., feeling disgusted by habitual behaviors) (4). In stroke populations, self-disgust is significantly more prevalent than in the general population. Its occurrence is closely associated with post-stroke neurobiological alterations, such as dysregulation of neurotransmitters including serotonin, as well as changes in body image, functional impairments, and loss of social and family roles. Severe self-disgust has been associated with exacerbation of depressive and anxiety symptoms, increased risk of suicide, and greater vulnerability to personality-related psychopathology. It may also impair the ability to adapt to stroke sequelae and diminish quality of life during rehabilitation (5, 6).

Social integration refers to the process by which individuals establish and maintain social connections through occupational participation, interpersonal relationships, community engagement, and leisure activities. Adequate social integration enhances self-worth, promotes subjective wellbeing, facilitates the development of healthy behavioral patterns, and contributes to improved quality of life (7).

Self-efficacy, defined as an individual's subjective judgment of their ability to successfully perform specific behaviors, plays a central role in stroke rehabilitation. In stroke patients, self-efficacy is primarily reflected in their confidence in completing rehabilitation exercises, restoring activities of daily living, and managing their condition (8). Evidence suggests that higher self-efficacy significantly improves adherence to rehabilitation training, encouraging more active and sustained participation in therapy (9). Moreover, stronger self-efficacy promotes greater initiative and persistence in functional recovery, particularly in motor function and daily living abilities, thereby contributing to better overall functional outcomes (10).

In the *Outline of the* “*Healthy China 2030*” *Plan* (2016), China incorporated public health into the national strategic development framework, emphasized strengthening health promotion systems, implementing comprehensive chronic disease prevention and control, expanding rehabilitation services for individuals with disabilities, and enhancing national health literacy through lifestyle education (11). In line with these national priorities, comprehensive stroke rehabilitation should extend beyond clinical management to include health education, with the goal of improving disease-related knowledge and fostering effective health beliefs.

The present study investigated the mediating role of self-efficacy in the relationship between social integration and self-disgust among patients with stroke, aiming to elucidate the mechanisms underlying these variables. By focusing on the enhancement of self-management ability, this study aims to inform strategies for preventing complications, reducing recurrence, optimizing rehabilitation, and ultimately improving quality of life.

## 2 Materials and methods

### 2.1 Study participants

A convenience sampling method was employed to recruit patients with stroke who attended the Department of Encephalopathy in a tertiary hospital in Changsha between January and October 2024. Participants were enrolled in a questionnaire survey.

Inclusion criteria: 1. Age ≥ 18 years; 2. Diagnosis of stroke according to the Key Points in the Diagnosis of Various Major Cerebrovascular Diseases in China (2019) (12), confirmed by MRI or head CT; 3. Clear consciousness with adequate reading and comprehension ability; 4. Clinically stable condition; 5. Presence of motor dysfunction or facial abnormalities; 6. Provision of informed consent and voluntary participation.

Exclusion criteria: 1. History of diagnosed mental disorders prior to stroke; 2. Presence of severe organic diseases involving major organs; 3. Concurrent enrollment in other interventional clinical studies.

This study was reviewed and approved by the institutional Ethics Committee (Approval No.: HN-LL-SWJW-2023-048).

### 2.2 Methods

#### 2.2.1 General information questionnaire

A self-designed questionnaire was used to collect demographic and clinical data, including sex, age, educational level, marital status, occupational status, self-care ability, number of hospitalizations, treatment methods, sleep status, and stroke type.

#### 2.2.2 Stroke self-efficacy questionnaire (SSEQ)

The SSEQ was originally developed by Jones et al. (13) to assess rehabilitation self-efficacy in patients with stroke. The scale originally contained 13 items. The Chinese version, revised by Li et al. (14), consists of 11 items. Responses are rated on a 11-point Likert scale (0–10), yielding a total score range of 0–110, with higher scores indicating stronger self-efficacy. In the study by Li et al., the Cronbach's α coefficient of the SSEQ was 0.879, demonstrating its good (or excellent) internal consistency.

#### 2.2.3 Berkman-Syme social network index (BSSNI)

The BSSNI, a widespread tool used in epidemiological studies, was used to evaluate the level of social integration (15). It comprises four dimensions: marital status (married/partnered = 3, divorced/widowed/separated/single = 0); participation in religious or belief-based activities (0–3 points according to frequency); participation in social organizations (0–3 points according to frequency); and number of friends (0–3 points based on quantity). Total scores range from 0 to 12 are classified into four levels: Grade I (0–5), Grade II (6), Grade III (7–8), and Grade IV (9–12). Higher scores indicate stronger social integration.

#### 2.2.4 Questionnaire for the assessment of self-disgust (QASD)

The QASD was originally developed by Schienle et al. (16), and later revised to a Chinese version by Jin et al. (17). It comprises two dimensions: personal disgust (9 items) and behavioral disgust (5 items). Each item is rated on a 5-point Likert scale ranging from 0 (“very inconsistent”) to 4 ("very consistent”). Total scores range from 0 to 56, with higher scores indicating greater severity of self-disgust. In this study, the Cronbach's α coefficient was 0.836.

### 2.3 Data collection

Data were collected by one graduate nursing student who had received systematic training in survey administration. To ensure consistency in the data collection process, this data collector received standardized training, which covered the study objectives, questionnaire content, standardized instructions for participants, and appropriate approaches for addressing patients' questions.

Prior to the formal survey, the research team explained the study background, objectives, and questionnaire requirements to participants and their legal guardians. Written, informed consent was obtained after confirming their understanding. To ensure data quality, all questionnaires were administered following standardized instructions and collected on-site immediately upon completion. Any ambiguous responses were clarified with participants immediately to ensure completeness. Questionnaires with contradictory or irregular responses were excluded as invalid. This study includes 13 independent variables; therefore, after accounting for an estimated 20% rate of invalid questionnaires, the necessary sample size ranges from 156 to 312 participants. During the study period (January to October 2024), A total of 240 stroke patients were admitted to the Department of Encephalopathy at our hospital. Among them, 13 patients were excluded due to impaired consciousness, dementia, or severe aphasia, the presence of other major neurological or psychiatric disorders, or concurrent enrollment in other interventional clinical studies. As a result, 227 met the final eligibility criteria. All eligible patients were invited to participate, and 220 patients who provided written informed consent were ultimately enrolled in the study. Finally, 207 valid responses were returned, yielding an effective response rate of 94%. A study flow diagram following the STROBE statement has been shown in Supplementary Figure 1.

### 2.4 Statistical analysis

Data were analyzed using SPSS version 25.0. Categorical variables are presented as frequencies and percentages, and continuous variables as medians with interquartile ranges. Continuous variables were first tested for normality using the Shapiro-Wilk test. The results showed that the main variables, including social integration, self-efficacy, and self-disgust, were approximately normally distributed (Shapiro-Wilk test, *p* > 0.05). As these variables were continuous and normally distributed, Pearson's correlation analysis was used to examine relationships among self-efficacy, social integration, and self-disgust.

Preliminary correlational analyses were performed to assess the linear relationships among study variables and inform the subsequent mediation model. Mediation analysis was conducted using Mplus 8.7, and the significance of indirect effects was assessed with the Bootstrap method.

## 3 Results

Among the 207 participants with stroke, 117 (56.5%) were male and 90 (43.5%) were female (Table 1). The associations between demographic factors with social integration, self-efficacy, and self-disgust among these stroke patients are shown in Supplementary Table 1. No significant associations between demographic factors with social integration, self-efficacy, and self-disgust were observed. Pearson's correlation analysis demonstrated that self-disgust was negatively correlated with social integration (*r* = −0.235, *p* < 0.01), self-efficacy was positively correlated with social integration (*r* = 0.237, *p* < 0.01), and self-efficacy was negatively correlated with self-disgust (*r* = −0.621, *p* < 0.01; Table 2). The total scores of self-efficacy, social integration, and self-disgust are presented in Table 3.

**Table 1:** Demographic and clinical characteristics of participants (*n* = 207).

Variable	Number (%)	Variable	Number (%)
Age (years)	–	Self-care ability	–
• < 40	14 (6.8)	Mild	103 (49.8)
•≥41–65	83 (40.1)	Moderate	83 (40.1)
•≥65	110 (53.1)	Complete	21 (10.1)
Marital status		Treatment method	–
• Married	171 (82, 6)	Conservative treatment	153 (73.9)
• Divorce	8 (3.9)	Thrombolytic therapy	54 (26.1)
• Widowed	27 (13.0)	Number of hospitalizations	–
• Single	1 (0.5)	0–1	10 (4.8)
Occupational status	–	2–3	101 (48.8)
• Employed	21 (10.2)	>3	96 (46.4)
• Retired	64 (30.9)	Sleep quality	–
• Unemployed	122 (58.9)	General	96 (43.4)
Education level		Good	74 (35.7)
• Primary school	49 (23.6)	Poor	37 (17.9)
• Junior high school	78 (37.7)	Stroke type	–
• Senior high school	30 (14.5)	Cerebral infarction	157 (75.8)
• College or above	50 (24.2)	Cerebral hemorrhage	50 (24.2)

**Table 2:** Correlation analysis of self-efficacy, social integration, and self-disgust (*r, n* = 207).

Variable	Social integration	Self-disgust	Self-efficacy
Social integration	1	–	–
Self-disgust	−0.235^**^	1	–
Self-efficacy	0.237^**^	−0.621^**^	1

^**^indicates statistical significance at the 0.01 level (*p* < 0.01).

**Table 3:** Total and item scores of self-efficacy, social integration, and self-disgust (*n* = 207).

Variable	Total score	Item score
Social integration	70 (67, 77)	6.36 (6.09, 0.64)
Self-disgust	40 (36, 45)	2.8 (2.6, 3.6)
Self-efficacy	3 (2, 5)	0.75 (0.50, 1.25)

**Figure 1:**
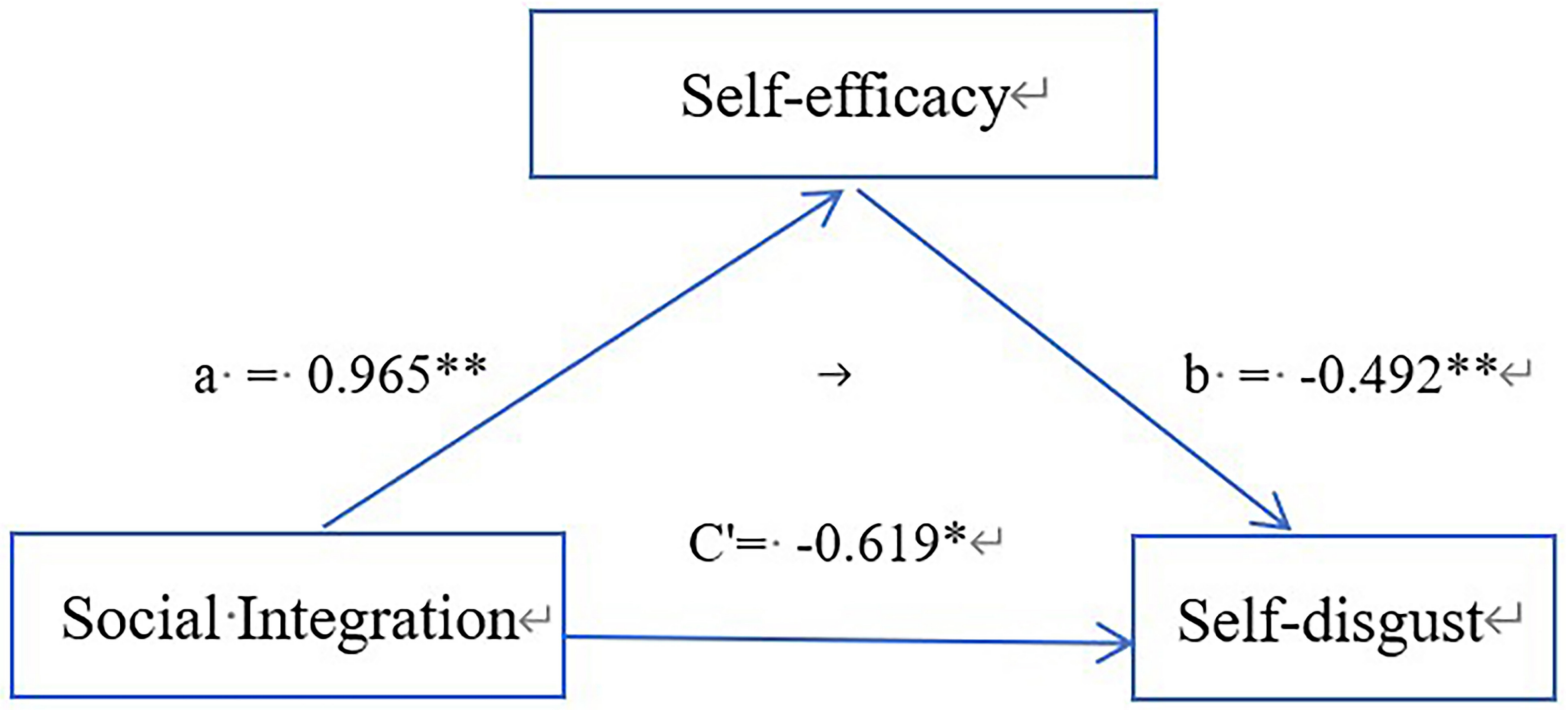
Mediating role of self-efficacy in the relationship between social integration and self-disgust among patients with stroke. ^*^p < 0.05, ^**^p < 0.001.

Mediation analysis indicated a significant total effect of social integration on self-disgust [β = −1.094, 95% CI: (−1.568, −0.620), *p* < 0.001]. Social integration also exerted a significant direct effect on self-disgust [β = −0.619, 95% CI: (−1.026, −0.213), *p* < 0.001]. In addition, self-efficacy demonstrated a significant direct effect on self-disgust [β = −0.475, 95% CI: (−0.744, −0.236), *p* < 0.001]. A mediating effect was considered statistically significant when the 95% confidence interval (CI) did not include zero. The mediating effect model of self-efficacy between social integration and self-disgust is presented in Figure 1 and detailed results are provided in Table 4.

**Table 4:** Mediating effect analysis of self-efficacy between social integration and self-disgust in patients with stroke.

Mediation effect test results	Effect value	SE	T	95% confidence interval		% Effect of total
				LLCI	ULCI	
Total effect	−1.094	0.24	−4.553	−1.568	−0.620	–
Direct effect	−0.619	0.206	−3.007	−1.026	−0.213	56.58
Indirect effect	−0.475	0.13	–	−0.744	−0.236	43.42

The total effect value = −1.094, with 95% confidence interval [−1.568, −0.620], which does not include 0, indicating a significant total effect.

## 4 Discussion

In this study, we examined the relationships among social integration, self-efficacy, and self-disgust in patients with stroke, and tested the mediating role of self-efficacy between social integration and self-disgust, providing evidence for the development of targeted psychological interventions and social support strategies. A total of 207 stroke patients were included into the final analysis. The results showed that self-disgust was at a moderately high level, social integration was slightly below average, and self-efficacy was at a moderate level. Social integration was significantly negatively correlated with self-disgust and significantly positively correlated with self-efficacy, while self-efficacy was significantly negatively correlated with self-disgust. Mediation analysis further indicated that self-efficacy partially mediated the relationship between social integration and self-disgust, with an indirect effect of −0.118, accounting for 16.01% of the total effect, and the mediation effect was statistically significant.

### 4.1 Current status of self-efficacy, social integration, and self-disgust in patients with stroke

The median self-disgust score among patients with stroke was 40 (36–45), indicating a moderately high level. This may be attributed to post-stroke brain injury, particularly impairments in neurotransmitter systems involved in emotional regulation, such as serotonin dysregulation, which can heighten negative self-perception and emotional distress (18). In this way, social engagement may counteract the effects of neurotransmitter dysregulation, reducing self-disgust and enhancing confidence in one's abilities, thereby integrating psychosocial and neurological mechanisms in our model of post-stroke recovery. Additionally, compared with the pre-stroke state, patients often experience significant life changes, including reduced self-care ability, increased dependence on others, diminished social participation, restricted social activities, altered self-perception and body image, loss of previous social roles, disruptions of established lifestyles. Unfavorable prognoses may further necessitate long-term reliance on assistive devices or caregivers, increasing the likelihood of pessimism and low self-esteem (19). These results highlight the importance of strengthening psychological interventions, fostering comprehensive support networks that include hospitals, families, and communities.

The median social integration score was 3 (2–5), indicating a moderately low level. Physical limitations, psychological challenges, and societal barriers continue to hinder successful integration. These findings underscore the need for comprehensive social support systems that respect and address the unique needs of patients with stroke, safeguard their legal rights, and ensure equitable access to education, employment, welfare, and healthcare resources.

The median self-efficacy score was 70 (67–77), indicating a moderate level, consistent with findings reported by Li et al. (20). Inadequate or inappropriate rehabilitation exercise may hinder functional recovery, perpetuating low self-efficacy in a negative feedback cycle. To address this, healthcare professional should implement individualized, evidence-based rehabilitation measures, conduct regular psychological assessments, and offer counseling to foster sustained improvements in self-efficacy.

### 4.2 Correlations between social integration, self-disgust, and self-efficacy in patients with stroke

This study identified a negative correlation between social integration and self-disgust in patients with stroke, consistent with the findings of Tan et al. (21). Self-disgust, characterized by negative emotional experiences such as self-deprecation, may be intensified by post-stroke motor impairments that limit mobility and language deficits that hinder communication. These limitations may create emotional distance from others, reduce opportunities to confide in family and friends, and reinforce self-disgust due to difficulties in accepting altered physical appearance.

In contrast, social integration was positively correlated with self-efficacy, a finding that aligns with results reported by Li et al. (22). Research on self-efficacy has been increasing both domestically and internationally; however, few studies have examined the mediating role of self-efficacy between social integration and self-stigmatization in stroke patients. Therefore, this study used a questionnaire survey to explore the mediating effect of self-efficacy on the relationship between social integration and self-stigmatization in stroke patients. According to social integration theory, individuals are not passive recipients of societal acceptance; rather, they actively participate in and adapt to sociocultural systems and organizational processes (23). Personal initiative and positivity play a crucial role in social integration. Therefore, encouraging opportunities for active social participation may enhance self-efficacy and facilitate adaptation to post-stroke challenges. Healthcare professionals should encourage patients with stroke to engage in social activities, provide positive reinforcement, and support confidence building as part of the rehabilitation process.

### 4.3 Mediating role of self-efficacy between social integration and self-disgust

The findings of this study demonstrate that self-efficacy partially mediates the relationship between social integration and self-disgust in patients with stroke. Higher levels of self-efficacy enable patients to adopt a more objective perspective of their condition, strengthen treatment confidence and compliance, and promote active participation in disease management. Prior research has identified exercise self-efficacy as a critical motivator for initiating and maintaining physical activity (24). Improved exercise self-efficacy enhances recognition of the value of rehabilitation exercise, reduces psychological barriers, and supports greater participation in activity, which in turn fosters positive self-cognition and self-perception.

The present findings further suggest that enhanced social integration can indirectly reduce self-disgust by strengthening self-efficacy. Conversely, limited or negative social integration may undermine self-efficacy and, subsequently, intensify self-disgust. These findings underscore the importance of developing interventions that simultaneously promote social integration and enhance self-efficacy to improve psychological wellbeing during stroke rehabilitation.

### 4.4 Limitations

The limitations of the present study should be acknowledged. First, the cross-sectional design precludes the establishment of causal relationships or determination of the temporal sequence among the variables included in the mediation model. Therefore, the observed mediation effects should be interpreted as associations rather than evidence of causal pathways. Second, demographic factors were not incorporated as covariates in the mediation model because they showed no significant associations with the primary study variables in the current sample. Nevertheless, residual confounding cannot be completely excluded, and future studies with larger and more diverse cohorts should incorporate relevant demographic and clinical characteristics to further evaluate the robustness of the observed mediation effect. Third, all participants were recruited from a single medical center, which may introduce single-site sampling bias related to patient characteristics, clinical management patterns, and referral practices. Consequently, the findings may not be fully generalizable to stroke populations from other regions, healthcare settings, or institutions with different patient profiles. Future multicenter studies involving diverse healthcare centers are warranted to validate the reproducibility and external validity of the identified associations and mediation pathways.

## 5 Conclusion

This study suggests that self-efficacy may partially mediate the relationship between social integration and self-disgust in stroke patients. This finding implies that enhancing self-efficacy and promoting social integration may reduce self-disgust and support rehabilitation.

## Data Availability

The raw data supporting the conclusions of this article will be made available by the authors, without undue reservation.

## Supplementary material

Supplementary Figure 1 Flowchart of Patient Enrollment.
