# Correction: Mediating role of self-efficacy in the relationship between social integration and self-disgust among patients with stroke

**DOI:** 10.3389/fneur.2026.2015040

**Published:** 2026-10-15

**Authors:** Cai-Dan Liu, Huan-Wen Luo, Yan-Lan Yu, Yun-Bo Zhou, Jia-Yi Zeng

**Affiliations:** 1 Department of Neurology, The First Affiliated Hospital of Hunan University of Chinese Medicine, Changsha, Hunan Province, China; 2 Nursing department, The First Affiliated Hospital of Hunan University of Chinese Medicine, Changsha, Hunan Province, China; 3 Hunan University of Chinese Medicine, Changsha, Hunan Province, China

**Keywords:** mediating effect, mental health, self-disgust, self-efficacy, social integration, stroke

In the published article, “The development and clinical validation research of a tailored traditional Chinese medicine cognitive enhancement program for patients with PSCI in the community, [22A0247]” was erroneously omitted from the **Funding** statement. The updated **Funding** statement appears below.

## Funding

The author(s) declared that financial support was received for this work and/or its publication. 1. National Key Specialized Discipline in Traditional Chinese Medicine - Nursing [State Traditional Chinese Medicine Administration Document (2024) No. 90]. 2. 2025 Annual Natural Science Foundation Project of Hunan Province (Number: 2025JJ80949). 3. Healthcare Research Project of Hunan Province in 2025 (Number: 20257255). 4. 2026 Annual Natural Science Foundation Project of Hunan Province (Number: 2026JJ81408). 5. Research Project of the Hunan Nursing Association: Development and Application of a Self-Management Program for High-Risk Stroke Patients Based on the COM-B Model (Number: HNKYY202505). 6. The development and clinical validation research of a tailored traditional Chinese medicine cognitive enhancement program for patients with PSCI in the community, (22A0247).

The original version of this article has been updated.

