# Supplementary material for: Mediating role of self-efficacy in the relationship between social integration and self-disgust among patients with stroke
Source: Front Neurol. 2026 Sep 11;17:1868193. doi: 10.3389/fneur.2026.1868193 (PMC13612949; doi:10.3389/fneur.2026.1868193)
Supplement: Supplementary file 1 [file Table_1.DOCX]

**Supplementary Table 1. Associations between demographic factors with social integration, self-efficacy, and self-disgust among stroke patients (N=207)**

| **Demographic**  **factors** | **Category** | **Number (%)** | **Self-efficacy (*p*-value)** | **social integration (*p*-value)** | **self-disgust (*p*-value)** |
| --- | --- | --- | --- | --- | --- |
| **Sex** | Male | 148 (71.5) | 0.351 | 0.739 | 0.915 |
|  | Female | 59 (28.5) |  |  |  |
| **Age (years)** | <40 | 14 (6.8) | 0.481 | 0.323 | 0.329 |
|  | 40-65 | 83 (40.1) |  |  |  |
|  | >65 | 110 (53.1) |  |  |  |
| **Marital status** | Married | 171 (82.6) | 0.602 | 0.379 | 0.780 |
|  | Divorced | 8 (3.9) |  |  |  |
|  | Widowed | 27 (13) |  |  |  |
|  | Single | 1 (0.5) |  |  |  |
| **Education** | Elementary school | 49 (23.7) | 0.522 | 0.669 | 0.968 |
|  | Junior high school | 78 (37.7) |  |  |  |
|  | High school | 30 (14.5) |  |  |  |
|  | College | 50 (24.1) |  |  |  |
| **Occupation** | Employed | 20 (9.7) | 0.370 | 0.504 | 0.376 |
|  | Retired | 65 (31.4) |  |  |  |
|  | Unemployed | 122 (58.9) |  |  |  |
